# Circ_0003266 sponges miR-503-5p to suppress colorectal cancer progression via regulating PDCD4 expression

**DOI:** 10.1186/s12885-021-07997-0

**Published:** 2021-03-16

**Authors:** Caihong Wen, Xiaoqing Feng, Honggang Yuan, Yong Gong, Guangsheng Wang

**Affiliations:** 1 Department of Oncology, Yichang Central People’s Hospital, The First College of Clinical Medical Science, China Three Gorges University, NO.183 Yiling Avenue, Yichang, 443003 Hubei China; 2 Department of Urology Surgery, Yichang Central People’s Hospital, The First College of Clinical Medical Science, China Three Gorges University, Yichang, 443003 Hubei China; 3 Department of Digestive Internal, Yichang Central People’s Hospital, The First College of Clinical Medical Science, China Three Gorges University, Yichang, 443003 Hubei China; 4 Department of Gastrointestinal Surgery, Yichang Central People’s Hospital, The First College of Clinical Medical Science, China Three Gorges University, Yichang, 443003 Hubei China

**Keywords:** Colorectal cancer, circ_0003266, miR-503-5p, PDCD4

## Abstract

**Background:**

Circular RNAs (circRNAs) feature prominently in tumor progression. However, the biological function and molecular mechanism of circ_0003266 in colorectal cancer (CRC) require further investigation.

**Methods:**

Circ_0003266 expression in 46 pairs CRC tissues / adjacent tissues, and CRC cell lines was detected by quantitative real-time polymerase chain reaction (qRT-PCR); after circ_0003266 was overexpressed or knocked down in CRC cells, cell proliferation, apoptosis, migration, and invasion were evaluated by the cell counting kit-8 (CCK-8), flow cytometry, and Transwell assays, respectively; the interaction among circ_0003266, miR-503-5p, and programmed cell death 4 (PDCD4) was confirmed using bioinformatics analysis and dual-luciferase reporter assay; PDCD4 protein expression in CRC cells was quantified using Western blot.

**Results:**

Circ_0003266 was significantly lowly expressed in CRC tissues and cell lines. Circ_0003266 overexpression markedly repressed CRC cell proliferation, migration, and invasion, and accelerated the cell apoptosis, but its overexpression promoted the malignant phenotypes of CRC cells. PDCD4 was a direct target of miR-503-5p and circ_0003266 promoted PDCD4 expression by competitively sponging miR-503-5p.

**Conclusion:**

Circ_0003266 suppresses the CRC progression via sponging miR-503-5p and regulating PDCD4 expressions, which suggests that circ_0003266 may serve as a novel target for the treatment of CRC.

**Supplementary Information:**

The online version contains supplementary material available at 10.1186/s12885-021-07997-0.

## Background

Colorectal cancer (CRC) is a common cancer, and its mortality ranks among cancers worldwide, with nearly 900,000 deaths each year [1]. Although great progress has been made in the diagnosis and treatment in recent years, the therapeutic effect of some patients with CRC is not good due to high frequency of metastasis and recurrence [2]. Besides, the incidence of CRC is increasing among people under 45 years old [3]. The research on circular RNAs (circRNAs), microRNA (miRNA), and their regulatory mechanism in CRC may provide novel diagnostic biomarkers and therapeutic targets for CRC, which may help improve the prognosis of the patients with CRC [4, 5].

CircRNA is a class of non-coding RNA derived from the reverse splicing of the precursor mRNA, which is with a covalent closed-loop structure formed by splicing the 5′ end of one exon with the 3′ end of another exon [6]. Many circRNAs are dysregulated in diverse diseases [7, 8]. For example, hsa_circ_0013958 expression is up-regulated in lung adenocarcinoma tissues, cells, and plasma of the patients, which is positively correlated with TNM stage and lymphatic metastasis; functionally, circ_0013958 accelerates the proliferation and invasion of lung adenocarcinoma cells and inhibits the apoptosis [9]. Some circRNAs are abnormally expressed in CRC tissues, in which circRNAs with down-regulated expression levels, such as circ_0008287, circ_0069865, and circVPS13–1, are tumor suppressors, while circRNAs with up-regulated expression levels, such as circMGAT5, circ_0000724, and circAATF-1, act as tumor promoters [10]. However, the function of circ_0003266 in CRC awaits further study.

MicroRNAs (miRNAs), an endogenous RNA with about 20–22 nt, is involved in regulating many physiological and pathological processes. CircRNAs can exert their function via sponging miRNA, competitively combine with the corresponding miRNA through base pairing, and regulate gene expression at the post-transcriptional level [11]. For example, circAPLP2 activates Notch signaling pathway in CRC by targeting miR-101-3p, thus promoting tumor proliferation and metastasis [12]. CircAGFG1 drives the metastasis of CRC by modulating the YY1/CTNNB1 axis via sponging miR-4262 and miR-185-5p [13]. Reportedly, miR-503 promotes the migration and invasion of CRC cells by regulating programmed cell death 4 (PDCD4) [14]. However, whether miR-503/PDCD4 axis is involved in a competitively endogenous RNA (ceRNA) network in CRC is still obscure.

In this work, we used circRNA microarray to identify the abnormal expression of circRNAs in CRC tissues. We demonstrated that, circ_0003266 expression was significantly down-regulated in CRC. Functionally, circ_0003266 impeded the proliferation and metastasis of CRC cells and promoted apoptosis by regulating miR-503-5p/PDCD4 pathway.

## Methods

### Tissue samples

The study enrolled 46 CRC patients (22 males and 24 females, aged from 23 to 60 years) recruited between 2018 and 2019 from the Yichang Central People’s Hospital. All CRC patients who had undergone surgery without chemotherapy or radiotherapy were diagnosed by pathological examination. The cancerous and paracancerous tissues (more than 2 cm from the edge of the tumor) were collected and immediately stored in liquid nitrogen. Tumor histological grading and staging were performed according to the World Health Organization classification criteria and the Tumor Node Metastasis system. This study was endorsed by the Institutional Ethics Committee of Yichang Central People’s Hospital, and written informed consents were obtained from all patients before the research.

### Expression profile analysis of circRNAs

CircRNA expression profile data were downloaded from Gene Expression Omnibus (GEO) database (http://www.ncbi.nlm.nih.gov/geo/). Using keywords (“circRNA” and “colorectal cancer”) in the GEO database, we searched the circRNAs microarray related with CRC and found the dataset GSE142837. We used GEO2R online analysis tool to get log fold change and adjusted *P*-value. Excel was used to screen out the circRNAs with *P* < 0.05 and |log_2_fold change (FC)| > 1 in CRC tissues (v.s. non-tumor tissues).

### Cell culture

Human normal colonic epithelial cells (NCM460) and CRC cell lines (HT29, SW480, HCT-116, Lovo, and DLD-1) were obtained from the Cell Bank of the Chinese Academy of Sciences (Shanghai, China). All CRC cells were cultured in Dulbecco’s Modified Eagle’s Medium (DMEM, Invitrogen, Carlsbad, CA, USA) with 10% fetal bovine serum (FBS, Gibco, Carlsbad, CA, USA), 100 U/ml penicillin and 100 μg/ml streptomycin (Sigma, St. Louis, MO, USA) at 37 °C in 5% CO_2_.

### Cell transfection

The overexpression vectors of circ_0003266 and PDCD4 were constructed using pcDNA3.1 vector. siRNA targeting circ_0003266 (si-circ_0003266), miR-503-5p mimic and inhibitor, and their corresponding controls were purchased from GenePharma (Shanghai, China). The negative control (si-NC or miR-control) was adopted as the control vectors. The above mentioned oligonucleotides or plasmids (50 nM) and Lipofectamine™ 2000 reagents (Invitrogen, Carlsbad, CA, USA) were diluted using 100 μL of Opti-MEM medium (Invitrogen, Carlsbad, CA, USA), respectively, and incubated for 2 min at room temperature. Then they were mixed and incubated at room temperature for 20 min. The mixture was then added to a 6-well plate (containing 3 × 10^5^ cells/well). 48 h after the transfection, the transfection efficiency was detected.

### Quantitative real-time polymerase chain reaction (qRT-PCR)

Total RNA was isolated using TRIzol (Vazyme, Nanjing, China). cDNA synthesis was conducted using the TaqMan MicroRNA reverse transcription kit (Applied Biosystems, Foster City, CA) for miR-503-5p and PrimeScript RT Master Mix Kit (Takara Biotechnology Co., Ltd., Dalian, China) were used for preparing the cDNA to detect PDCD4 and circ_0003266. Then quantitative PCR was performed, and circ_0003266 and PDCD4 expression levels were determined by SYBR SYBR Premix Ex Taq II (Takara, Dalian, China), and miR-503-5p expression was quantified by stem-loop primer SYBR Green qRT-PCR (Synbio Tech, Suzhou, China). GAPDH and U6 worked as internal controls for circRNA/mRNA and miRNA, respectively. qRT-PCR was operated on an ABI 7500 Fast Real-Time PCR System (Applied Biosystems, Waltham, MA, UK), and relative expression levels were calculated by 2^−ΔΔCT^ method. Primer sequences are listed in Table 1.

**Table 1:** Sequences used for qRT-PCR

Name	Primer sequences
circ_0003266	Forward: 5′-AGTTGACAGCGGTACCATCC-3′
	Reverse: 5′-TGTAGGTTCGGCAAGTCCTC-3′
miR-503-5p	Forward:5′-CCTATTTCCCATGATTCCTTCATA-3’
	Reverse:5′-GTAATACGGTTATCCACGCG-3′
U6	Forward:5′-ATTGGAACGATACAGAGAAGATT-3’
	Reverse:5′-GGAACGCTTCACGAATTTG-3’
PDCD4	Forward: 5′-ACAGGTGTATGATGTGGAGGA-3′
	Reverse: 5′-TTCTCAAATGCCCTTTCATCCAA-3′
GAPDH	Forward:5′-AACGGATTTGGTCGTATTGGG-3’
	Reverse:5′-CCTGGAAGATGGTGATGG GAT-3’

### RNase R resistance analysis of circRNAs

To confirm the circular property of circ_0003266, 2 μg of total RNAs was treated with or without 3 U/mg RNase R (Epicentre Technologies, Madison, WI, USA) for 30 min at 37 °C in RNase R reaction buffer. Then the expression level of circ_0003266 was detected by qRT-PCR.

### Western blotting

Total protein was extracted by RIPA lysis buffer (Beyotime, Shanghai, China). Subsequently, protein concentration was measured using a bicinchoninic assay. Then 30 μg of protein / lane was separated via 10% SDS-PAGE and then transferred onto a PVDF membrane (Millipore, Schwalbach, Germany). After being blocked with TBS containing 5% skimmed milk at room temperature for 1 h, the membrane was incubated with rabbit anti-PDCD4 antibody (1:1000, ab51495, Abcam, Cambridge, UK) and anti-GAPDH antibody (1:2000; ab37168, Abcam, Cambridge, UK), respectively at 4 °C overnight, and then incubated with HRP-conjugated secondary antibodies (1:5000, Beyotime, Shanghai, China) at room temperature for 1 h. GAPDH was used as the internal control. Ultimately, the protein bands were developed by the enhanced chemiluminescence reagent (Beyotime, Shanghai, China).

### Cell counting kit-8 (CCK-8) assay

Cells were harvested 24 h after the transfection. A total of 1 × 10^3^ CRC cells was transferred into each well of the 96-well plates and CCK-8 assay was performed every 24 h. Briefly, 10 μL of CCK-8 solution (Sigma, St. Louis, MO, USA) was added into each well at the corresponding time points, and the cells were cultured for another 1 h. The the viability of the cells (indicated by the value of absorbance) was analyzed at a wavelength of 450 nm, using a microplate reader (Potenov, Beijing, China). 4 d later, the proliferation of the cells in each group was plotted, with the absorbance values as the ordinate, and the time as the abscissa.

### Transwell assay

Cell migration and invasion assays were performed using Transwell chambers (Corning Corning, NY, USA). In cell migration assay, a total of 5 × 10^5^ cells was suspended in serum-free medium and transferred into the upper chamber of each Transwell insert, while the lower chamber was added with 600 μL of complete medium with 20% FBS. After the culture for 24 h, cells in the upper surface of the filter were removed with cotton swabs and cells remaining on the bottom surface of the filter were fixed with methanol at room temperature for 15 min, followed by being staining with 1% crystal violet at room temperature for 30 min. Finally, stained cells were photographed under a light microscope at × 200 magnification and, and the number of these cells of five randomly selected fields was counted. In cell invasion assay, the filter was pre-coated with diluted Matrigel, and the other procedures were executed as described above.

### Flow cytometry

Cell apoptosis was detected by Annexin V-FITC Apoptosis Detection Kit (Sigma, St. Louis, MO, USA). Transfected cells were centrifugated at 5000×g for 5 min at room temperature. Cell pellets were rinsed with PBS and re-suspended in the staining buffer. Then the cells were stained with 5 μL of propodium iodide staning solution in the dark for 30 min at 4 °C and subsequently stained with 5 μL of Annexin V-FITC staining solution for 20 min at room temperature. After that, apoptotic cells were analyzed by a flow cytometer (BD Biosciences, San Jose, CA, USA).

### Dual-luciferase reporter assay

The wild type (WT) fragments of circ_0003266 / PDCD4 3′-untranslated region (UTR) containing the predicted binding sites of miR-503-5p, and mutant (MUT) circ_0003266/PDCD4 3’UTR sequences were provided by GenePharma Co., Ltd. (Shanghai, China). The fragments were cloned into the pGL3 Dual-Luciferase miRNA Target Expression vector (Promega, Madison, WI, USA), according to the manufacturer’s protocol. The miR-503-5p mimic or negative control mimic was co-transfected into HEK-293 T cells with the wild-type or mutant reporter vectors. After 48 h, the relative activity of luciferase was determined using the Dual-Luciferase Reporter Assay kit (Promega, Madison, WI, USA) in line with the manufacturer’s instructions.

### Statistical analysis

Graphs were generated by GraphPad Prism 8.0 (GraphPad Software, Inc., La Jolla, CA, USA), and statistical analysis was performed with SPSS 22.0 (IBM, Chicago, IL, USA.). Student’s *t*-test or one-way analysis of variance was adopted for making the comparisons. Pearson correlation analysis was conducted to analyze the correlations between the two indicators. Chi-square test was used to analyze the association between circ_0003266 expression and the clinical characteristics of the patients. *P* < 0 .05 was considered statistically significant.

## Results

### Circ_0003266 is lowly expressed in CRC

**Fig. 1:**
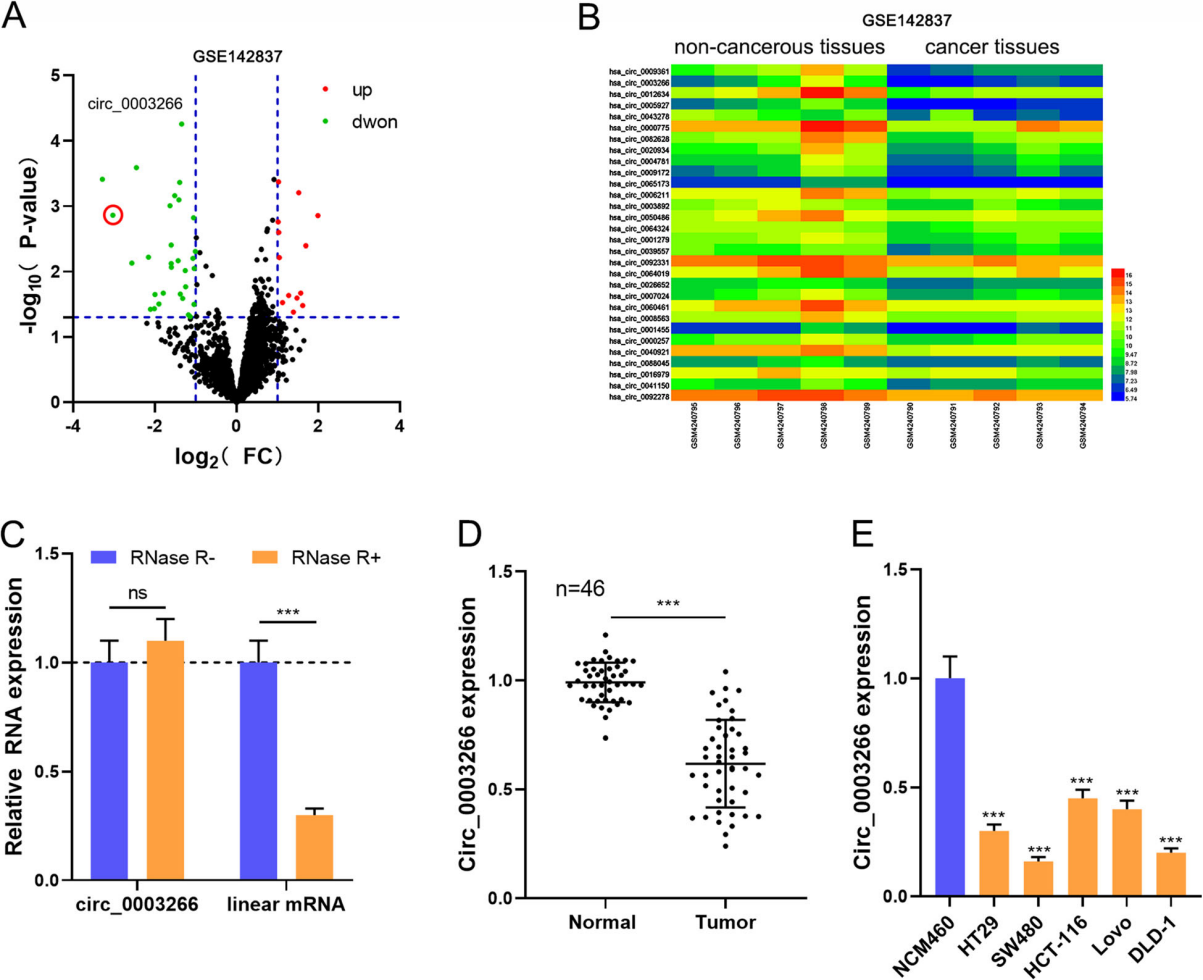
Circ_0003266 was lowly expressed in CRC. **a**. Volcano plot showed the up-regulated and down-regulated circRNAs in CRC tissues (v.s. non-cancerous tissues) from the analysis of GSE142837. Highly-expressed circRNAs were indicated by “red”, and the lowly-expressed circRNAs were indicated by “green”. **b**. Heat map showed the down-regulated circRNAs in CRC tissues v.s. non-cancerous tissues from the analysis of GSE142837. **c**. qRT-PCR was used to detect the expression levels of circ_0003266 and GAPDH after the treatment of RNase R. **d**. qRT-PCR was used to detect the expression of circ_0003266 in human CRC tissues and adjacent tissues. **e**. qRT-PCR was used to detect the expression of circ_0003266 in normal colonic epithelial cells (NCM460) and CRC cell lines (HT29, SW480, HCT-116, Lovo, and DLD-1). ***P < 0.001

The expression profile of circRNAs in 5 pairs of CRC tissue and normal tissue samples were analyzed by circRNA microarray (GSE142837). Out of 3292 circRNAs, 43 circRNAs (|log_2_FC| > 1, *P* < 0.05) were screened out, among which the expression levels of 13 circRNAs were up-regulated and 30 were down-regulated (Fig. 1a-b). In this study, circ_0003266, whose expression was significantly down-regulated, was investigated. CircRNAs have no free ends, showing a longer half-life and resistance to RNase R, compared with liner RNA. To verify the circular structure of circ_0003266, RNase R resistance assay was performed, and it was found that only linear mRNA (GAPDH) expression level was decreased after RNA R treatment, implying that circ_0003266 has a loop structure (Fig. 1c). Next, we found that circ_0003266 expression in CRC tissues was markedly lower than that in adjacent tissues by qRT-PCR (Fig. 1d). Besides, circ_0003266 expression in CRC cell line was significantly down-regulated compared with that in NCM460 cells (Fig. 1e). Furthermore, we analyzed the relationship between the expression of circ_0003266 and the clinicopathological features of CRC patients. The results illustrated that circ_0003266 expression level was negatively correlated with tumor grade and stage of CRC patients (Table 2). These dates suggested that the abnormal down-regulation of circ_0003266 expression might affect CRC progression.

**Table 2:** The relationship between the expression of circ_0003266 and clinical features of CRC

Expression of circ_0003266				
Pathological factors	Low(23)	High(23)	chi-square value	*P* value
Sex			0.3485	0.555
Male	10	12		
Female	13	11		
Age			0.0897	0.765
< 60	14	13		
≥ 60	9	10		
Lymph node metastasis			0.8070	0.369
Absent	15	12		
Present	8	11		
Grade			5.5758	0.018^*^
Low	7	15		
High	16	8		
T stage			4.2933	0.038^*^
T1-T2	9	16		
T3-T4	14	7		

### Circ_0003266 suppresses the malignant phenotypes of CRC cells

**Fig. 2:**
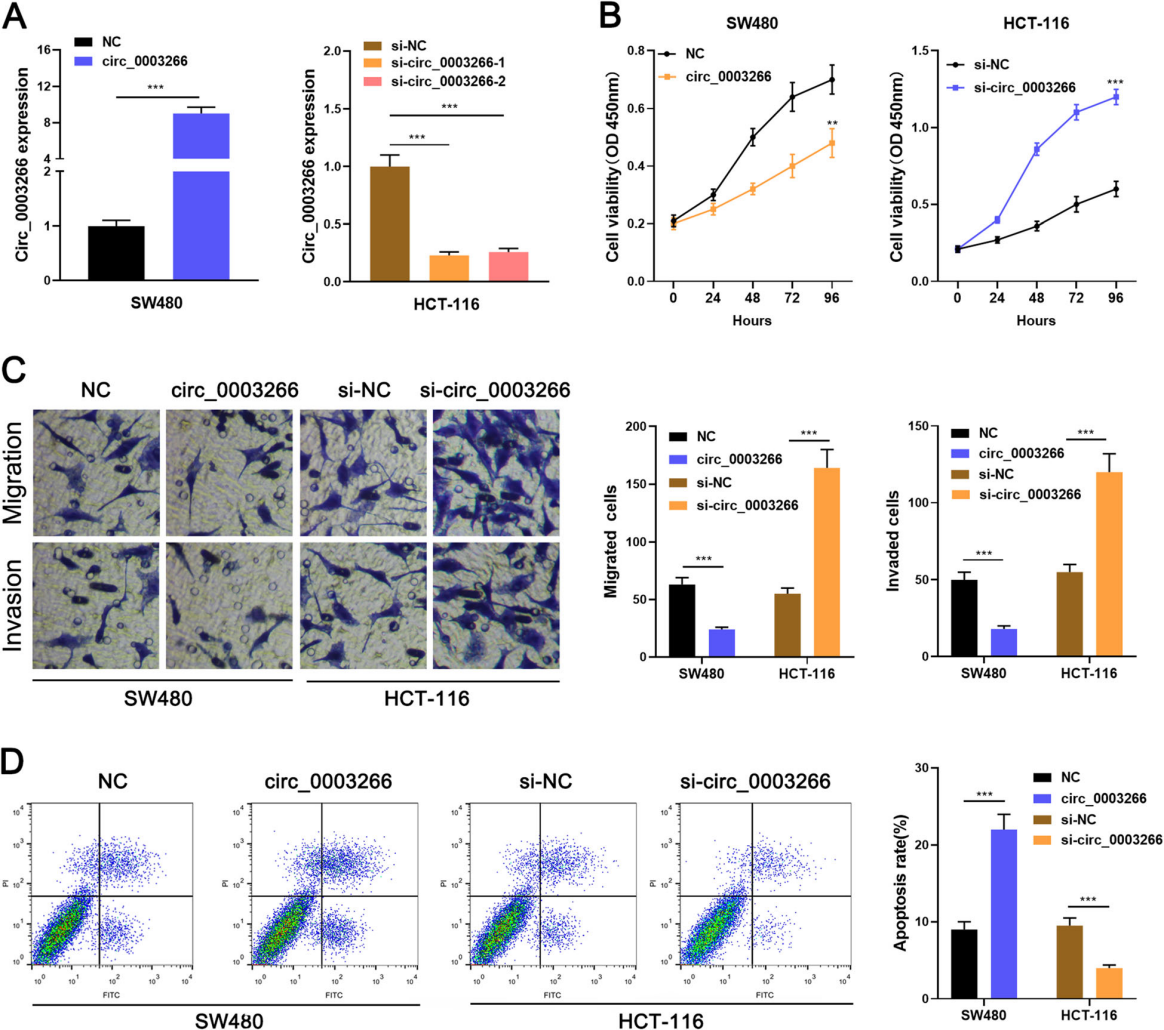
Circ_0003266 inhibited the proliferation and metastasis of CRC cells, and promoted the apoptosis. **a**. qRT-PCR was employed to detect the expression of circ_0003266 in SW480 and HCT-116 cells transfected with circ_0003266 or two independent siRNAs targeting circ_0003266. **b**. CCK8 assay was used to detect the effects of circ_0003266 on the proliferation capability of SW480 and HCT-116 cells. **c**. Transwell assay was used to detect the effects of circ_0003266 on migration and invasion capability of SW480 and HCT-116 cells. **d**. Flow cytometry was used to detect the effects of circ_0003266 on the apoptosis rate of SW480 and HCT-116 cells. **P < 0.01 and ***P < 0.001

Circ_0003266 overexpression plasmid was constructed and transfected into SW480 cells, and si-circ_0003266 was transfected into HCT-116 cells. The transfection efficacy was confirmed by qRT-PCR (Fig. 2a). To further clarify the function of circ_0003266 in CRC, CCK-8, flow cytometry, and Transwell assays were performed, and the results displayed that circ_0003266 overexpression significantly decreased the growth, migration, and invasion of SW480 cells, and expedited the apoptosis, but circ_0003266 silencing functioned oppositely on HCT-116 cells (Fig. 2 b-d). These results showed that circ_0003266 was a tumor suppressor in CRC.

### Circ_0003266 negatively regulates miR-503-5p expression

**Fig. 3:**
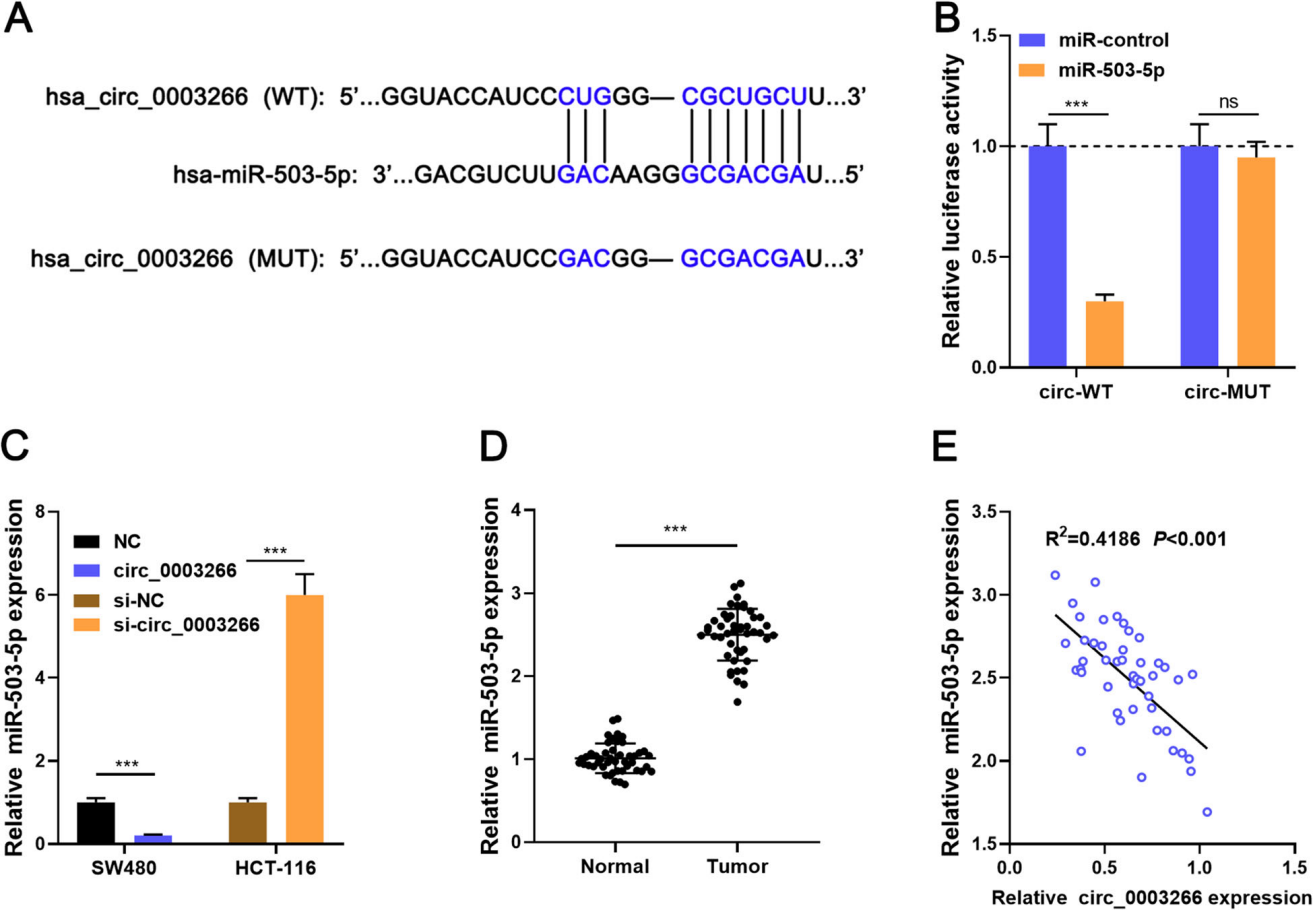
MiR-503-5p was a direct target of circ_0003266. **a**. Circular RNA Interactome was used to predict the binding site between circ_0003266 and miR-503-5p. **b**. Dual-luciferase reporter gene assay was used to confirm the relationship between circ_0003266 and miR-503-5p. **c**. qRT-PCR was used to detect the expression of miR-503-5p in SW480 and HCT-116 cells transfected with NC, circ_0003266 overexpression plasmids, si-NC, or si-circ_0003266. **d**. qRT-PCR was used to detect the expression of miR-503-5p in human CRC tissues and adjacent tissues. **e**. Pearson correlation analysis showed the negative relationship between circ_0003266 and miR-503-5p in CRC tissues. ***P < 0.001

We analyzed the candidate miRNAs with complementary sequences to circ_0003266 using the online CircInteractome database, and found that miR-503-5p was one of the potential targets (Fig. 3a). Dual-luciferase reporter gene assay uncovered that miR-503-5p could decrease the luciferase activity of circ_0003266-WT reporter but had no significant effect on circ_0003266-MUT reporter (Fig. 3b). Besides, qRT-PCR results showed that circ_0003266 overexpression resulted in a significant decrease in miR-503-5p expression in CRC cells, while the depletion of circ_0003266 worked oppositely (Fig. 3c). Besides, miR-503-5p expression in CRC tissues was higher than that in adjacent tissues and negatively correlated with circ_0003266 expression (Fig. 3 d-e). These evidence indicated that miR-503-5p was the target of circ_0003266.

### MiR-503-5p negatively regulates PDCD4 expression

**Fig. 4:**
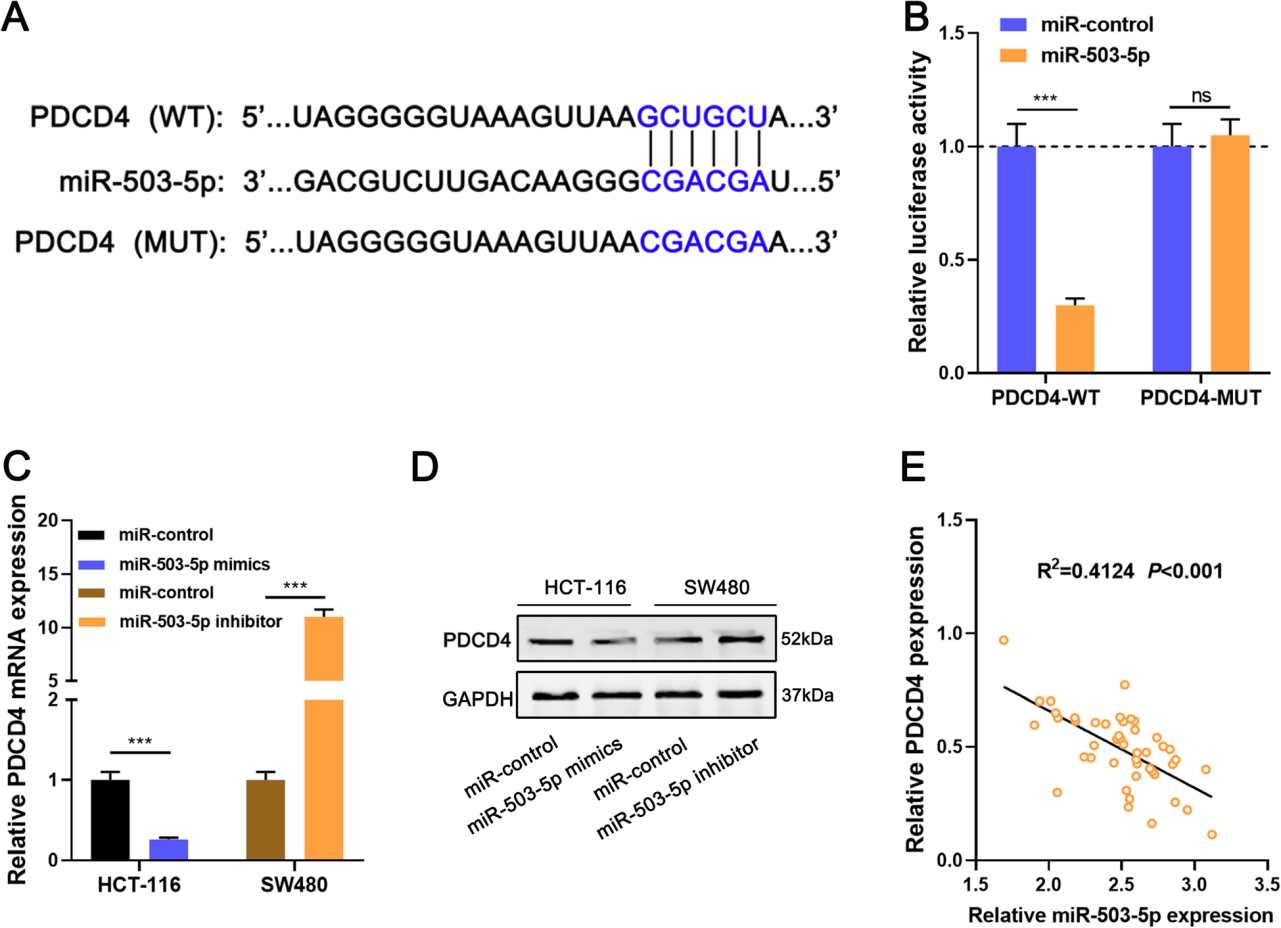
PDCD4 was confirmed as a target gene of miR-503-5p. **a**. StarBase was used to predict the binding site of miR-503-5p and PDCD4. **b**. Dual-luciferase reporter gene assay was used to confirm the relationship between miR-503-5p and PDCD4. **c-d**. qRT-PCR and Western blot were used to detect the expression of PDCD4 in CRC cells after miR-503-5p was modulated. **e**. Pearson correlation analysis showed the negative relationship between PDCD4 and miR-503-5p in CRC tissues. ***P < 0.001

Through analyzing StarBase databse, TargetScan database, and miRDB database, we found that there was a binding site in PDCD4 3’UTR to miR-503-5p (Fig. 4a), which is consistent with the previous report [14]. Dual-luciferase reporter gene assay indicated that miR-503-5p overexpression markedly repressed the luciferase activity of PDCD4-WT reporter but did not exert an impact on that of PDCD4-MUT reporter (Fig. 4b). MiR-503-5p mimics significantly inhibited PDCD4 mRNA and protein expression levels while miR-503-5p inhibitors worked oppositely (Fig. 4c-d). Meanwhile, there was a negative correlation between the expression levels of miR-503-5p and PDCD4 in CRC tissues (Fig. 4e).

### Circ_0003266 restrains CRC progression via modulating miR-503-5p/PDCD4 axis

**Fig. 5:**
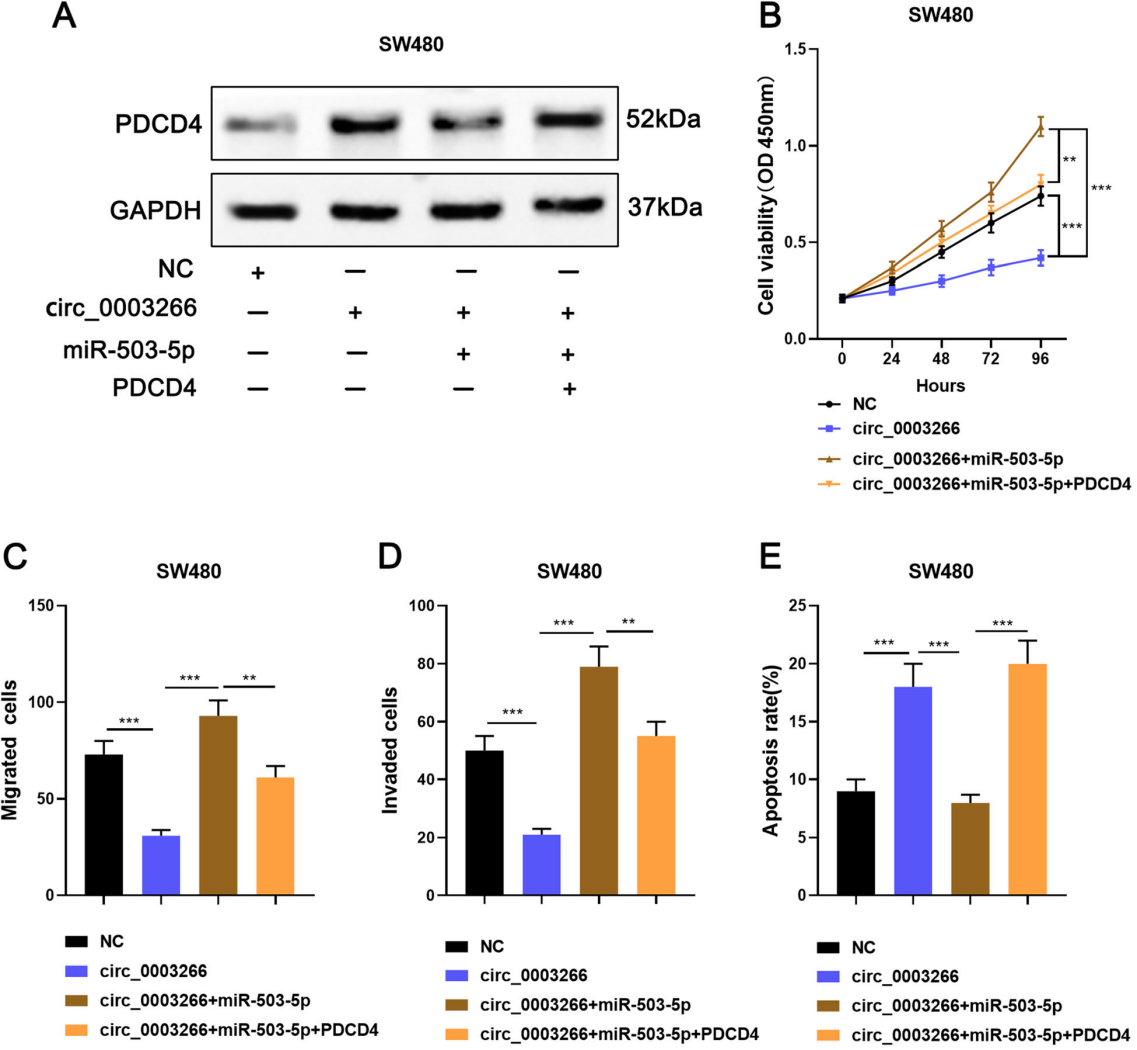
Circ_0003266 inhibited malignant phenotypes of CRC cells by regulating miR-503-5p/PDCD4 axis. **a**. With transfection, SW480 cells were divided into four groups: NC group, circ_0003266 overexpression group, circ_0003266 overexpression + miR-503-5p overexpression group, and circ_0003266 overexpression + miR-503-5p overexpression + PDCD4 overexpression group, and Western blot assay was used to detect the expression of PDCD4 in SW480 cells. **b**. CCK8 assay was used to detect the proliferation of SW480 cells after the transfection. **c-d**. Transwell assay was used to detect the migration and invasion of SW480 cells after the transfection. **e**. Flow cytometry was used to detect the apoptosis rate of SW480 cells after the transfection. **P < 0.01 and ***P < 0.001

To pinpoint whether circ_0003266 exerted its functions via modulating miR-503-5p/PDCD4 pathway, we performed rescue assays. The SW480 cells were divided into four groups: NC group, circ_0003266 overexpression group, circ_0003266 overexpression + miR-503-5p overexpression group, and circ_0003266 overexpression + miR-503-5p overexpression + PDCD4 overexpression group. Western blot assay showed that, circ_0003266 significantly promoted the expression of PDCD4 in SW480 cells, and the co-transfection of miR-503-5p reversed this effects, and the transfection of PDCD4 overexpression plasmids counteracted the effects of miR-503-5p (Fig. 5a). Furthermore, functional assays showed that, circ_0003266 overexpression inhibited the proliferation, migration, and invasion, and promoted the apoptosis of SW480 cells; however, miR-503-5p mimics totally reversed these effects (Fig. 5b-e); additionally, PDCD4 overexpression reversed the effects induced by miR-503-5p overexpression (Fig. 5b-e). These findings demonstrated that circ_0003266 suppressed CRC via modulating miR-503-5p/PDCD4 pathway.

## Discussion

CircRNAs are discovered in RNA viruses as early as the in the 1970s, and in recent year, multiple circRNAs are identified in the transcriptome of human cells [15]. Reportedly, circRNAs are more stable and abundant than linear RNA, and circRNAs are mainly located in the cytoplasm and they have miRNA response elements; what’s more, circRNAs have other biological functions such as working as the scaffold in the assembly of protein complexes, regulating alternative splitting, modulating RNA-protein interactions, and so on [15–18]. CircRNAs are implicated in regulating the pathogenesis of human diseases including diabetes, nervous system diseases, cardiovascular diseases, and cancers, etc. [19]. For example, CirchHipk3 expression is observably raised in CRC tissues and cell lines, and functionally, CirchHipk3 knock-down can markedly impede the growth, migration, and invasion of CRC cells [20]. Circ-ITGA7 inhibits CRC cell proliferation via adsorbing miR-3187-3p and increasing ASXL1 expressions [21]. Here, we found that circ_0003266 expression was significantly down-regulated in CRC. Additionally, circ_0003266 restrained the proliferation and metastatic potential of CRC cells, and expedited the apoptosis. Our results suggested that it could probably be a biomarker and therapy target for CRC.

MiRNAs regulate mRNA expression by inhibiting translation or promoting degradation, and they are important regulators in cancer biology [22]. For example, the decreased expression of miR-4319, as reported, is related to the poor prognosis of CRC patients, and miR-4319 significantly inhibits the proliferation of CRC cells and changes cell cycle distribution by targeting ABTB1 [23]. Reportedly, circRNAs act as miRNAs sponges to regulate tumor progression. For example, circ_0136666 accelerates the multiplication and invasion of CRC cells via miR-136/SH2B1 axis [24]. In this work, we identified miR-503-5p as the target miRNA of circ_003266 by bioinformatics analysis and dual-luciferase reporter gene assay. MiR-503-5p is abnormally expressed in various cancers including hepatocellular carcinoma, ovarian cancer, cervical cancer, and oral squamous cell carcinoma [25–28]. Besides, miR-503-5p expression in CRC is significantly increased, which expedites the migration and invasion of CRC cells [14]. In this work, we observed that miR-503-5p expression was elevated in CRC tissues, and miR-503-5p promoted proliferation and metastasis of CRC cells, and inhibited the apoptosis, which is consistent with findings of the previous research [14]. Moreover, miR-503-5p could counteract the inhibitory effects of circ_0003266 on CRC procession. These findings suggested that circ_0003266 contributed to the dysregulation of miR-503-5p in CRC, and its function was dependent on miR-503-5p.

PDCD4 is a tumor suppressor, and its expression is frequently down-regulated in various types of cancers [29]. PDCD4 protein is composed of 469 amino acid residues, and PDCD4 binds to eIF4A and restrains its helicase activity [30–32]. PDCD4 expression is abnormally down-regulated in CRC, and PDCD4 represses the translation of Sin1 translation via interacting the eIF4A, and inhibits CRC progression [30]. PDCD4 also directly combines with mRNA of c-Myb, Bcl-xL, and XIAP to suppress their translation, thereby inhibiting cell proliferation and promoting apoptosis [32]. Previous studies report that PDCD4 inhibits the progression of several cancer cells, including hepatocellular carcinoma, breast cancer, and melanoma [33–35]. Reportedly, miR-503-5p can target PDCD4 [14]. In this study, we further explored the impact of circ_0003266 on PDCD4, the results of which demonstrated that circ_0003266 could positively regulate PDCD4 via adsorbing miR-503-5p.

There are some limitations of the present work. Firstly, our findings are only based on in vitro experiments, and in vivo assays can further confirm the role of circ_0003266 in CRC progression in the future. Secondly, the relationship between circ_0003266 and the prognosis of the CRC patients is still obscure, and survival analysis of more patients with follow-up information should be performed in the future to evaluate the prognostic value of circ_0003266. Lastly, CircInteractome database also predicts other miRNAs, which can probably be regulated by circ_0003266, and whether circ_0003266 could regulate CRC progression via modulating these miRNAs should be explored.

## Conclusion

Circ_0003266 is lowly expressed in CRC tissues and cells. Mechanistically, circ_0003266 inhibits CRC progression via modulating PDCD4 expressions by acting as ceRNA of miR-503-5p. The findings of the present study highlight the potential role of circ_0003266 as a tumor suppressor in CRC, which provides a novel therapeutic target for CRC treatment.

## Supplementary Information

**Additional file 1.**

## Data Availability

The data used to support the findings of this study are available from the corresponding author upon request.

## Abbreviations

CircRNAs: Circular RNAs
CRC: Colorectal cancer
qRT-PCR: Quantitative real-time polymerase chain reaction
PDCD4: Programmed cell death 4
CCK-8: Cell counting kit-8
ceRNA: Competitive endogenous RNA
miRNA: MicroRNA
FBS: Fetal bovine serum
siRNAs: Small interference RNAs
PVDF: Polyvinylidene fluoride
PBS: Phosphate buffered saline
