# Supplementary figures and images for: Circ_0003266 sponges miR-503-5p to suppress colorectal cancer progression via regulating PDCD4 expression
Source: BMC Cancer. 2021 Mar 16;21:284. doi: 10.1186/s12885-021-07997-0 (PMC7968268; doi:10.1186/s12885-021-07997-0)

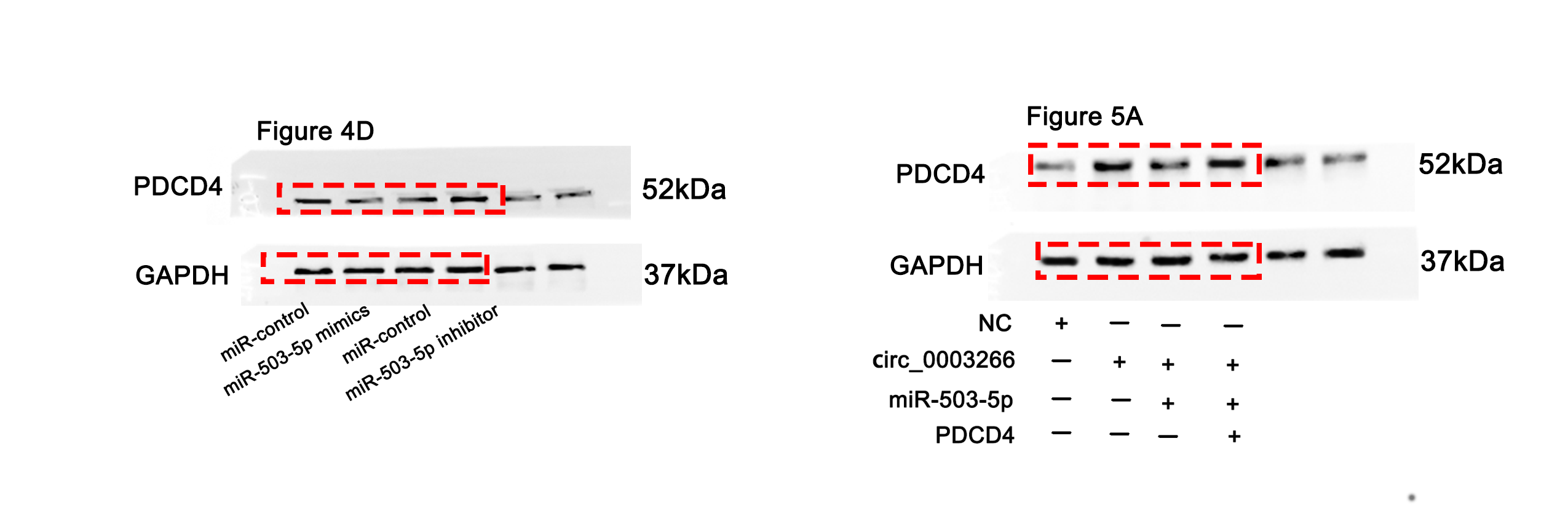

Supplement: Supplementary file 1 — Additional file 1. [file 12885_2021_7997_MOESM1_ESM.tif]
